# Cooperation, decision time, and culture: Online experiments with American and Indian participants

**DOI:** 10.1371/journal.pone.0171252

**Published:** 2017-02-23

**Authors:** Akihiro Nishi, Nicholas A. Christakis, David G. Rand

**Affiliations:** 1 Department of Epidemiology, UCLA Fielding School of Public Health, Los Angeles, California, United States of America; 2 Yale Institute for Network Science, Yale University, New Haven, Connecticut, United States of America; 3 Department of Sociology, Yale University, New Haven, Connecticut, United States of America; 4 Department of Ecology and Evolutionary Biology, Yale University, New Haven, Connecticut, United States of America; 5 Department of Medicine, Yale University, New Haven, Connecticut, United States of America; 6 Department of Psychology, Yale University, New Haven, Connecticut, United States of America; 7 Department of Economics, Yale University, New Haven, Connecticut, United States of America; University of Bristol, UNITED KINGDOM

## Abstract

Two separate bodies of work have examined whether culture affects cooperation in economic games and whether cooperative or non-cooperative decisions occur more quickly. Here, we connect this work by exploring the relationship between decision time and cooperation in American versus Indian subjects. We use a series of dynamic social network experiments in which subjects play a repeated public goods game: 80 sessions for a total of 1,462 subjects (1,059 from the United States, 337 from India, and 66 from other countries) making 13,560 decisions. In the first round, where subjects do not know if connecting neighbors are cooperative, American subjects are highly cooperative and decide faster when cooperating than when defecting, whereas a majority of Indian subjects defect and Indians decide faster when defecting than when cooperating. Almost the same is true in later rounds where neighbors were previously cooperative (a cooperative environment) except decision time among Indian subjects. However, when connecting neighbors were previously not cooperative (a non-cooperative environment), a large majority of both American and Indian subjects defect, and defection is faster than cooperation among both sets of subjects. Our results imply the cultural background of subjects in their real life affects the speed of cooperation decision-making differentially in online social environments.

## Introduction

Cooperation among non-kin is central to human societies [1]. Among many approaches to study cooperation, two that have received considerable attention in recent years are the exploration of cross-cultural differences and the investigation of decision times. A large body of evidence indicates that cooperation rates can vary substantially across countries and cultures [2–13]. In particular, a subject’s cooperation in economic game experiments is typically positively related to the quality of institutions under which that subject lives [3, 9–11], perhaps due (at least in part) to the observation that strong institutions foster the development of prosocial norms [10, 12].

A separate body of work has found that decision times correlate with choosing cooperation versus defection, with some studies finding that cooperation occurs more quickly [14–20] and others finding the opposite [21–23]. These seemingly contradictory results have been reconciled by an account of decision times based on conflictedness [24–27]: less conflicted decisions (where one option is strongly preferred over the other) occur quickly, whereas more conflicted decisions (where the difference in preference strength between the options is small) occur slowly. Therefore, in settings where cooperation is attractive to most people (and thus is chosen more frequently than defection), cooperators will tend to be less conflicted than defectors, and cooperation decisions will be faster than defection decisions. Conversely, in settings where defection is more attractive to people (and thus is chosen more frequently then cooperation), the opposite may be true. This can help explain why the correlation between decision time and cooperation varies across studies, and why it varies with social environment within repeated game studies (where reciprocal decisions are faster than non-reciprocal decisions [26]).

In the present study, we bring these two lines of work together and explore how the relationship between decision time and cooperation varies based on culture. We do so by comparing the behavior of subjects from the United States and India in an online repeated cooperation game involving dynamic social networks.

The United States and India offer an attractive comparison because subjects from these two countries can be recruited from the same online labor market (Amazon Mechanical Turk, MTurk [28]) but they differ substantially in terms of institutional quality (and in other ways, of course). For example, the United States was rated higher than India on all dimensions of the Rule of Law Index 2015 compiled by the World Justice Project (http://data.worldjusticeproject.org/), which considers constraints on government, absence of corruption, open government, fundamental rights, order and security, regulatory enforcement, civil justice, and criminal justice (overall rating on a 0–1 scale: United States, .73; India, .51). Furthermore, a 2015 report from Legatum Institute shows that, in terms of the level of social capital, the United States is 11^th^-ranked and India is 129^th^-ranked across 142 countries in the world [29]. For this and other reasons, we would expect American subjects to be more likely to develop norms of cooperation. Accordingly, American subjects have indeed been found to be more cooperative than Indian subjects in 1-shot games in prior work [30–33].

We focus on play in a repeated game, rather than a 1-shot game, because repeated play offers a richer set up behaviors to explore. As described above, recent work has shown that decision speed varies based on social environment within the game [26]. Thus we can examine how the *speed* of cooperation differs between American and Indian subjects not just in the absence of information about their partners’ play (as in 1-shot games), but also in the presence of specifically cooperative versus non-cooperative partners.

## Methods

### Data

We used the data from Nishi et al (2015) [34], in which 1,462 human subjects were recruited through Amazon Mechanical Turk [28] from all over the world for an experiment conducted in English. The main purpose of the data collection was to examine the effect of inequality and wealth visibility on cooperation and social welfare in dynamic social networks. The subjects participated in one of 80 online sessions between October and December 2013, and interacted repeatedly with connected neighbors in a public goods game over 10 rounds using their own computer. Their cooperation decision-making was dichotomous: cooperate with all connecting neighbors or defect against all of them. The benefit-cost ratio was 2. We obtained 13,560 decisions. At each round after the first, subjects were informed of the cooperation decisions of their connecting neighbors in the prior round. The Yale University Human Subjects Committee approved this study, and waived the need for written informed consent from the participants.

Since we recorded the Internet Protocol (IP) address of each subject (through https://freegeoip.net/json/), we could obtain information regarding where each subject resided at the time of the experiment. Subjects came predominantly from the United States (N = 1,059, 72.4%), followed by India (N = 377, 25.8%). Others come from 29 different countries, but all sample sizes are less than 10 per country (and thus they are omitted from further analysis). Please note that the experimental conditions for those from the United States and those from India are exactly the same (except for the time of day, given the time zone difference—though we conducted experiments at all times of day and night, thus having subjects in all time periods), and that subjects never knew the country of origin of their connecting neighbors in the experiments. Information on socio-demographic factors such as age, sex, race/ethnicity, and country of birth was not collected.

### Measures

One of our main outcome variables was decision time [15, 20, 31, 35–38]. We used the same definition of decision time that we previously defined for our experimental setting [26]: the time between when a step in which each subject was asked to choose cooperate or defect appeared on the screen and when each subject clicked A (for cooperate) or B (for defect) on the screen. As recommended by prior literature [20], the subjects in the experiment were not informed that decision time was recorded. The median decision time is 3.85 seconds (interquartile range [IQR] = 2.63–6.66) for all the subjects, 3.77 seconds (IQR = 2.58–6.61) for the American subjects, and 4.07 seconds (IQR = 2.80–6.86) for the Indian subjects.

### Analytic procedure

Since our data regarding decision-making events was observed multiple times in a single subject and in a single session, we took into account the three-level hierarchical data structure using a multilevel mixed-effects logistic regression model for cooperation decision (binary outcome), and a multilevel mixed-effects linear regression for decision time (continuous outcome) (i.e., a random intercepts model of the multilevel analysis framework with restricted maximum likelihood [REML]) [39].

First, we examined the difference in cooperation rate between subjects from the United States and those from India at the first round (*unknown environment*, where subjects do not know if connecting neighbors are generally cooperative or not), in a *cooperative environment* (cooperation rate of connecting neighbors at the previous round ≥ 0.5) and in a *non-cooperative environment* (a rate < 0.5) at the later rounds (2^nd^ to 10^th^ rounds), separately. We use the same threshold for the cooperative environment (i.e. 0.5) as in prior work [26].

Second, we examined decision time. As in previous studies [15, 26, 31], we log_10_-transformed decision time (seconds) due to the skewness of the decision time distribution. The point estimates obtained were exponentiated back with the base of 10 to show the percent change in decision time for cooperation decisions as compared with defection decisions (as in [26]). For the data in the unknown environment, we examined the decision time difference between cooperation and defection separately among subjects from the United States and those from India, and jointly analyzed the data to calculate the interaction *P* value. For the data regarding the second round or later, we separately analyzed the data by the type of environment (cooperative or non-cooperative).

In the random intercepts model that we used, we added a continuous variable of round number as a covariate for the later-round analysis, because decision time naturally becomes shorter over rounds [26]. As a sensitivity analysis for the later-round analysis on decision time, we added 8 indicator variables representing the third to tenth rounds (the second round as a reference category), which did not substantially change the results. All *P* values reported below are based on the mixed-effects regression model.

## Results

**Fig 1:**
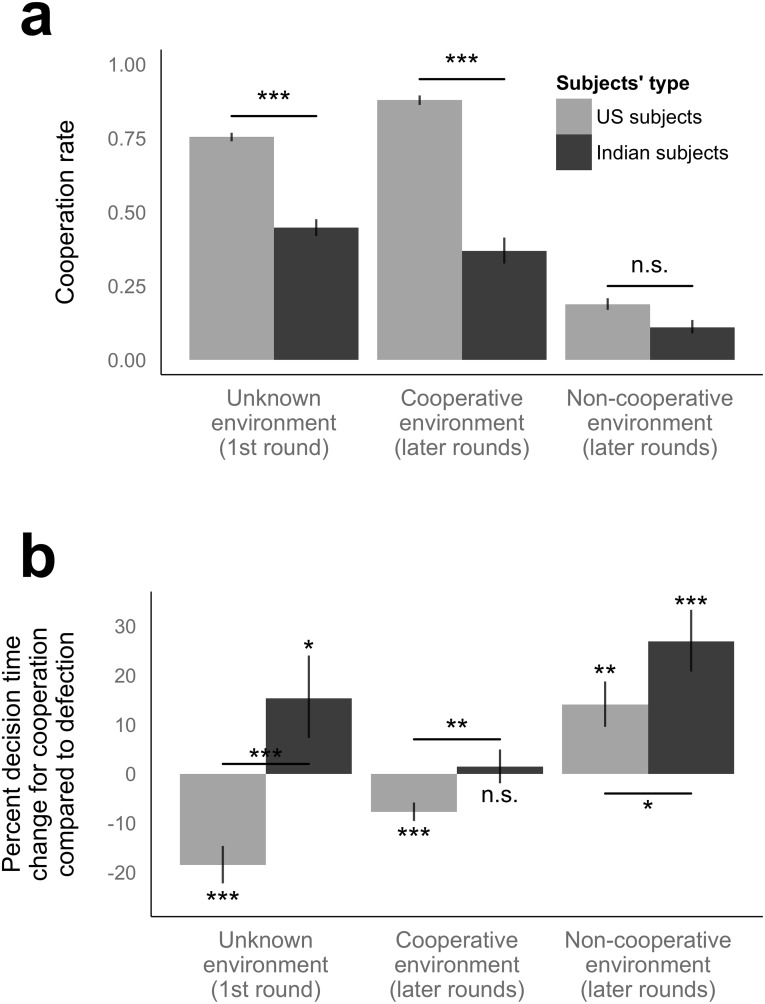
Cooperation takes less time than defection in unknown and cooperative environments and more time in a cooperative environment among subjects from the United States, while the patterns are different among subjects from India. **(a)** The cooperation rate of subjects from the United States and from India is shown at each setting for the reference. **(b)** The percent change in decision time for cooperation as compared with that for defection is estimated from multilevel analysis. The interaction *P* values are also calculated. **Left**, the results at the 1^st^ round (subjects do not know if connecting neighbors are cooperative or not). **Middle**, the results of cooperative environments at later rounds (≥ 2). **Right**, the results of non-cooperative social environments at later rounds (≥ 2). A cooperative environment for cooperation rate of connecting neighbors at the last round of 0.5 or more, and a non-cooperative environment for that of less than 0.5. Error bars, point estimates ± standard errors. n.s. for *P* ≥ 0.05, * for *P* < 0.05, ** for *P* < 0.01, and *** for *P* < 0.001.

First, we report the cooperation rate of the subjects from the United States and India (Fig 1). In an unknown environment, the cooperation rate is 75.4% for those from the United States and 44.8% for those from India (*P* < 0.001) (Fig 1a, left). In a cooperative environment (i.e., after the first round), it is 88.0% for those from the United States and 36.9% for those from India (*P* < 0.001) (Fig 1a, middle), while, in a non-cooperative environment, it is 18.9% for those from the United States and 11.0% for those from India (*P* = 0.151) (Fig 1a, right). In sum, the American subjects are more cooperative than the Indian subjects across all the different environments in our experimental setting (although, unsurprisingly, this difference was much less pronounced in the non-cooperative environment where defection is normative based on the principle of reciprocity).

Second, we report the relationship between cooperation and decision time. In the first round (where subjects do not know if connecting neighbors are cooperative or non-cooperative; i.e., the unknown environment), there is a negative relationship between decision time and cooperation among the subjects from the United States (cooperation is 18.5% faster, *P* < 0.001), and a positive relationship among those from India (cooperation is 15.3% slower, *P* = 0.049) (Fig 1b, left). This difference between American and Indian subjects in the relationship between decision time and cooperation is itself significant (interaction *P* < 0.001).

When they know that connecting neighbors were cooperative in the previous round (cooperative environment), there is a negative relationship between decision time and cooperation among those from the United States (cooperation is 7.7% faster, *P* < 0.001), and no relationship among those from India (cooperation is 1.4% slower, *P* = 0.671) (Fig 1b, middle). Again, the pattern among those from the United States differs from that among those from India (interaction *P* = 0.004).

When they know that connecting neighbors were *not* cooperative in the previous round (non-cooperative environment), there is a positive relationship between decision time and cooperation both among those from the United States (cooperation is 14.1% slower, *P* = 0.001) and those from India (cooperation is 26.9% slower, *P* < 0.001) (Fig 1b, right). The positive relationship is significantly weaker, however, among those from the United States compared to those from India (interaction *P* = 0.042).

## Discussion

In our experimental setting, we found marked differences between American and Indian subjects in cooperation, and in the relationship between cooperation and decision time. The American subjects are likely to be initially cooperative, and to keep cooperating at later rounds as long as neighbors are cooperative, but to switch to defection when neighbors defect. As per the conflictedness account of decision times, cooperation is therefore faster in the first round and in cooperative environments at later rounds, while defection is faster in non-cooperative environments.

Indian subjects, conversely, are relatively unlikely to cooperate, even in the first round or in later rounds where neighbors are cooperative. Thus, again as predicted by the conflictedness account, defection is faster than cooperation for Indian subjects across all environments. The difference is not statistically significant in the cooperative environment, perhaps because the psychology of reciprocation increased the psychological pull of cooperation even for those who eventually wound up defecting.

This difference in cooperation and decision time between the American and Indian subjects may have a root in the difference in quality of their social environment. As discussed in the Introduction and prior literature [13, 29, 30, 32, 40], Indian subjects live in a society which institution places less emphasis on social capital as compared with the US—low trust in their daily life may lead them to defect even against “friends” in the experimental setting. Since the friends in the experimental setting are anonymous and once-in-a-lifetime, it may be easier for them not to cooperate even after they realize that their “friends” are cooperative at the later rounds, but to defect and to prioritize gaining their own wealth. Subjects’ preference on how to allocate their resource between self and others, which has been the main focus of Social Value Orientation [41, 42], and time spent for its decision-making can be influenced by quality of social environment [26, 32, 43].

Our results provide further evidence for the importance of cross-cultural differences in human cooperation. In particular, they support theories involving a positive relationship between typically advantageous behavior outside the economic game (e.g., strength of institutions and social capital in one’s country of residence) and play in the game context [44–49]. Our results also provide additional support for the conflictedness account of decision times [24–27] by showing that, across cultures, the relative speed of cooperation varies with the absolute level of cooperation a person faces in the game.

It is important to note that our discussion of decision times has been limited to *correlations* between decision time and cooperation, rather than experiments in which decision times are experimentally manipulated. Evidence suggests that *manipulations* of decision time are *not* related to conflictedness, but instead are related to the relative reliance on intuition versus deliberation [25]; moreover, a recent meta-analysis of 51 such manipulation studies shows a consistent positive effect of promoting intuition relative to deliberation on cooperation in 1-shot games [50]. Relatively little work has investigated how the effect of decision time manipulations varies across cultures [32, 40, 51], and doing so is an important direction for future work.

## Supporting information

S1 File Data file. (CSV)

S2 File Data analysis file. (DO)

## References

[1] Rand DG, Nowak MA. Human cooperation. Trends in cognitive sciences. 2013;17(8):413–25. 10.1016/j.tics.2013.06.003 23856025

[2] Yamagishi T. The Provision of a sanctioning system in the United States and Japan. Soc Psychol Quart. 1988;51(3):265–71.

[3] Herrmann B, Thoni C, Gachter S. Antisocial punishment across societies. Science. 2008;319(5868):1362–7. 10.1126/science.1153808 18323447

[4] Wu JJ, Zhang BY, Zhou ZX, He QQ, Zheng XD, Cressman R, et al Costly punishment does not always increase cooperation. Proc Natl Acad Sci U S A. 2009;106(41):17448–51. 10.1073/pnas.0905918106 19805085 PMC2765097

[5] Gachter S, Herrmann B, Thoni C. Culture and cooperation. Philosophical Transactions of the Royal Society B-Biological Sciences. 2010;365(1553):2651–61. 10.1098/rstb.2010.0135 PMC2936171 20679109

[6] Blake PR, McAuliffe K, Corbit J, Callaghan TC, Barry O, Bowie A, et al The ontogeny of fairness in seven societies. Nature. 2015;528(7581):258–61. 10.1038/nature15703 26580018

[7] Henrich J, Boyd R, Bowles S, Camerer C, Fehr E, Gintis H, et al In search of homo economicus: Behavioral experiments in 15 small-scale societies. American Economic Review. 2001;91(2):73–8.

[8] Henrich J, Boyd R, Bowles S, Camerer C, Fehr E, Gintis H, et al “Economic man” in cross-cultural perspective: Behavioral experiments in 15 small-scale societies. Behavioral and Brain Science. 2005;28:795–855. 10.1017/S0140525X05000142 16372952

[9] Henrich J, Ensminger J, McElreath R, Barr A, Barrett C, Bolyanatz A, et al Markets, religion, community size, and the evolution of fairness and punishment. Science. 2010;327(5972):1480–4. 10.1126/science.1182238 20299588

[10] Stagnaro MN, Arechar AA, Rand DG. From good institutions to good norms: Top-down incentives to cooperate foster prosociality but not norm enforcement. SSRN: http://ssrncom/abstract=2720585. 2016. 10.1016/j.cognition.2017.01.017 PMC5875418 28249658

[11] Ellingsen T, Herrmann B, Nowak MA, Rand DG, Tarnita CE. Civic capital in two cultures: The nature of cooperation in Romania and USA. SSRN: http://ssrncom/abstract=2179575. 2012.

[12] Peysakhovich A, Rand DG. Habits of virtue: Creating norms of cooperation and defection in the laboratory. Management Science. 2016;62(3):631–47.

[13] Fehr E, Hoff K, Kshetramade M. Spite and development. American Economic Review. 2008;98(2):494–9.

[14] Zaki J, Mitchell JP. Equitable decision making is associated with neural markers of intrinsic value. Proc Natl Acad Sci U S A. 2011;108(49):19761–6. 10.1073/pnas.1112324108 22106300 PMC3241792

[15] Rand DG, Greene JD, Nowak MA. Spontaneous giving and calculated greed. Nature. 2012;489(7416):427–30. Epub 2012/09/22. 10.1038/nature11467 22996558

[16] Lotito G, Migheli M, Ortona G. Is cooperation instinctive? Evidence from the response times in a public goods game. Journal of Bioeconomics 2013;15(2):123–33.

[17] Cappelen AW, Nielsen UH, Tungodden B, Tyran JR, Wengström E. Fairness is intuitive. SSRN: http://ssrncom/abstract=2430774. 2014.

[18] Nielsen UH, Tyran JR, Wengstrom E. Second thoughts on free riding. Econ Lett. 2014;122(2):136–9.

[19] Rand DG, Fudenberg D, Dreber A. It's the thought that counts: The role of intentions in noisy repeated games. J Econ Behav Organ. 2015;116:481–99.

[20] Rubinstein A. Instinctive and cognitive reasoning: A study of response times. Econ J. 2007;117(523):1243–59.

[21] Piovesan M, Wengstrom E. Fast or fair? A study of response times. Econ Lett. 2009;105(2):193–6.

[22] Fiedler S, Glockner A, Nicklisch A, Dickert S. Social Value Orientation and information search in social dilemmas: An eye-tracking analysis. Organizational Behavior and Human Decision Processes. 2013;120(2):272–84.

[23] Lohse J, Goeschl T, Diederich J. Giving is a question of time: Response times and contributions to a real world public good. University of Heidelberg Department of Economics Discussion Paper Series (566). 2014.

[24] Krajbich I, Bartling B, Hare T, Fehr E. Rethinking fast and slow based on a critique of reaction-time reverse inference. Nature communications. 2015;6:7455 10.1038/ncomms8455 26135809 PMC4500827

[25] Evans AM, Dillon KD, Rand DG. Fast but not intuitive, slow but not reflective: Decision conflict drives reaction times in social dilemmas. Journal of Experimental Psychology: General. 2015;144(5):951–66. 26413891 10.1037/xge0000107

[26] Nishi A, Christakis NA, Evans AM, O'Malley AJ, Rand DG. Social environment shapes the speed of cooperation in experiments with humans. Scientific reports. 2016;6:29622 10.1038/srep29622 27435940 PMC4951649

[27] Evans AM, Van de Calseyde PPFM. The effects of observed decision time on expectations of extremity and cooperation. J Exp Soc Psychol. 2017;68:50–9.

[28] Buhrmester M, Kwang T, Gosling SD. Amazon's Mechanical Turk: A new source of inexpensive, yet high-quality, data? Perspect Psychol Sci. 2011;6(1):3–5. 26162106 10.1177/1745691610393980

[29] Legatum Institute. 2015 Legatum Prosperity Index (http://www.li.com/activities/publications/2015-legatum-prosperity-index). 2015.

[30] Raihani NJ, Mace R, Lamba S. The effect of $1, $5 and $10 stakes in an online dictator game. PLoS ONE. 2013;8(8):e73131 10.1371/journal.pone.0073131 23951342 PMC3741194

[31] Rand DG, Peysakhovich A, Kraft-Todd GT, Newman GE, Wurzbacher O, Nowak MA, et al Social heuristics shape intuitive cooperation. Nature communications. 2014;5:3677 10.1038/ncomms4677 24751464

[32] Capraro V, Cococcioni G. Social setting, intuition, and experience in laboratory experiments interact to shape cooperative decision-making. Proc Roy Soc B. 2015. 10.1098/rspb.2015.0237 PMC4528537 26156762

[33] Capraro V, Jordan JJ, Rand DG. Heuristics guide the implementation of social preferences in one-shot Prisoner's Dilemma experiments. Scientific reports. 2014;4:6790 10.1038/srep06790 25348470 PMC4210943

[34] Nishi A, Shirado H, Rand DG, Christakis NA. Inequality and visibility of wealth in experimental social networks. Nature. 2015;526:426–9. 10.1038/nature15392 26352469

[35] Wansink B, Just DR, Payne CR. Mindless eating and healthy heuristics for the irrational. American Economic Review. 2009;99(2):165–9. 29505211 10.1257/aer.99.2.165

[36] Deary IJ, Liewald D, Nissan J. A free, easy-to-use, computer-based simple and four-choice reaction time programme: The Deary-Liewald reaction time task. Behav Res Methods. 2011;43(1):258–68. 10.3758/s13428-010-0024-1 21287123

[37] Kosinski RA. A literature revew on reaction time (http://biae.clemson.edu/bpc/bp/lab/110/reaction.htm). 2013.

[38] Zaki J, Mitchell JP. Intuitive prosociality. Curr Dir Psychol Sci. 2013;22(6):466–70.

[39] Graubard BI, Korn EL. Modelling the sampling design in the analysis of health surveys. Statistical methods in medical research. 1996;5(3):263–81. 8931196 10.1177/096228029600500304

[40] Capraro V, Corgnet B, Espin AM, Hernan-Gonzalez R. Deliberation favors social efficiency by helping people disregard their relative shares: Evidence from US and India. SSRN: http://ssrncom/abstract=2799850. 2016.

[41] Griesinger DW, Livingston JW. Toward a model of interpersonal motivation in experimental games. Behav Sci. 1973;18(3):173–88.

[42] Liebrand WBG. The effect of social motives, communication and group-size on behavior in an n-person multi-stage mixed-motive game. Eur J Soc Psychol. 1984;14(3):239–64.

[43] Gachter S, Herrmann B. Reciprocity, culture and human cooperation: previous insights and a new cross-cultural experiment. Philosophical transactions of the Royal Society of London Series B, Biological sciences. 2009;364(1518):791–806. 10.1098/rstb.2008.0275 19073476 PMC2689715

[44] Chudek M, Henrich J. Culture gene coevolution, norm-psychology and the emergence of human prosociality. Trends in cognitive sciences. 2011;15(5):218–26. 10.1016/j.tics.2011.03.003 21482176

[45] Van Lange PAM, De Bruin E, Otten W, Joireman JA. Development of prosocial, individualistic, and competitive orientations: theory and preliminary evidence. Journal of personality and social psychology. 1997;73(4):733 9325591 10.1037//0022-3514.73.4.733

[46] Tooby J, Cosmides L. The past explains the present: Emotional adaptations and the structure of ancestral environments. Ethology and sociobiology. 1990;11(4):375–424.

[47] Kiyonari T, Tanida S, Yamagishi T. Social exchange and reciprocity: confusion or a heuristic? Evol Hum Behav. 2000;21(6):411–27. 11146306 10.1016/s1090-5138(00)00055-6

[48] Tomasello M, Melis AP, Tennie C, Wyman E, Herrmann E. Two key steps in the evolution of human cooperation. Curr Anthropol. 2012;53(6):673–92.

[49] Bear A, Rand DG. Intuition, deliberation, and the evolution of cooperation. Proc Natl Acad Sci U S A. 2016. 10.1073/pnas.1517780113 PMC4743833 26755603

[50] Rand DG. Cooperation, fast and slow: Meta-analytic evidence for a theory of social heuristics and self-interested deliberation. Psychological Science. 2016;27(9):1192–206. 27422875 10.1177/0956797616654455

[51] Rand DG, Kraft-Todd GT. Reflection does not undermine self-interested prosociality. Front Behav Neurosci. 2014;8:300 10.3389/fnbeh.2014.00300 25232309 PMC4153292

